# The impact of spikes in handgun acquisitions on firearm-related harms

**DOI:** 10.1186/s40621-019-0212-0

**Published:** 2019-08-26

**Authors:** Hannah S. Laqueur, Rose M. C. Kagawa, Christopher D. McCort, Rocco Pallin, Garen Wintemute

**Affiliations:** Violence Prevention Research Program, Department of Emergency Medicine, University of California, 2315 Stockton Blvd., Sacramento, CA 95817 USA

**Keywords:** Firearm injury, Elections, Mass shootings, Handguns

## Abstract

**Background:**

Research has documented sharp and short-lived increases in firearm acquisitions immediately following high-profile mass shootings and specific elections, increasing exposure to firearms at the community level. We exploit cross-city variation in the estimated number of excess handgun acquisitions in California following the 2012 presidential election and the Sandy Hook school shooting 5 weeks later to assess whether the additional handguns were associated with increases in the rate of firearm-related harms at the city level.

**Methods:**

We use a two-stage modeling approach. First, we estimate excess handguns as the difference between actual handgun acquisitions, as recorded in California’s Dealer Record of Sales, and expected acquisitions, as predicted by a seasonal autoregressive integrated moving-average (SARIMA) time series model. We use Poisson regression models to estimate the effect of city-level excess handgun purchasing on city-level changes in rates of firearm mortality and injury.

**Results:**

We estimate there were 36,142 excess handguns acquired in California in the 11 weeks following the election (95% prediction interval: 22,780 to 49,505); the Sandy Hook shooting occurred in week 6. We find city-level purchasing spikes were associated with higher rates of firearm injury in the 52 weeks post-election: a relative rate of 1.044 firearm injuries for each excess handgun per 1,000 people (95% CI: 1.000 to 1.089). This amounts to approximately 290 (95% CI: 0 to 616) additional firearm injuries (roughly a 4% increase) in California over the year. We do not detect statistically significant associations for shorter time windows or for firearm mortality.

**Conclusion:**

This study provides evidence for an association between excess handgun acquisitions following high-profile events and firearm injury at the community level. This suggests that even marginal increases in handgun prevalence may be impactful.

**Electronic supplementary material:**

The online version of this article (10.1186/s40621-019-0212-0) contains supplementary material, which is available to authorized users.

## Background

Firearm ownership is a well-established risk factor for interpersonal, self-directed, and unintentional firearm harm (Anglemyer et al., 2014; Kellermann et al., 1993; Kellermann et al., 1992; Wiebe, 2003). At the ecological level, the prevalence of firearm ownership has also been found to be associated with higher firearm homicide and suicide rates (Miller et al., 2002; Miller et al., 2007; Siegel et al., 2013). Given increasing rates of firearm purchasing in the United States over the last decades, and the rising burden of firearm harm (the rate of firearm deaths reached a 20-year high in 2017 (National Center for Injury Control and Prevention, 2019)), it is important to gain a deeper understanding of the relationship between firearm acquisition and firearm-related harm.

Research has documented large and short-lived increases in firearm acquisitions immediately following high-profile mass shootings (Liu & Wiebe, 2019) and the election and reelection of President Obama (Depetris-Chauvin, 2015). Studdert el al. (2017) find significant spikes in handgun purchasing in California in the six weeks following the widely publicized mass shooting at Sandy Hook elementary school in Newtown, Connecticut (2012) and in San Bernardino, California (2015), totaling 53 and 41% more purchases than expected. The California trends correspond with national reports. While there is no national database of firearm purchasing records, Levine and McKnight (2017) show a proxy for purchases - National Instant Criminal Background Check System (NICS) checks performed on individuals seeking to purchase a firearm through a licensed dealer - increased in the months immediately following the Sandy Hook mass shooting. The elections of President Obama in 2008 and 2012 were also followed by spikes in NICS checks (Depetris-Chauvin, 2015).

Based on previous research linking firearm ownership and firearm prevalence with increased risk of firearm harm, the sudden and unanticipated influx of firearms in a concentrated area such as a city could result in increases in firearm harm. Levine and McKnight found increases in firearm purchasing following the Sandy Hook school shooting were associated with increases in the number of unintentional firearm deaths at the state level, Levine & McKnight (2017). Another study found states with larger purchasing spikes following the 2008 presidential election were 20% more likely to experience a mass shooting event (Depetris-Chauvin, 2015). Associations with firearm injury, far more common than firearm mortality, have not been tested.

In the present study, we estimate whether the spike in handgun acquisitions in California following the 2012 presidential election and the Sandy Hook school shooting that took place five weeks after was associated with increases in fatal and non-fatal firearm injury. Assuming these historical events do not influence community-level firearm violence by avenues other than increasing handgun purchasing, variation in the degree of excess handgun purchasing across cities offers an opportunity to estimate independent associations between firearm acquisition and firearm-related harms at the community level.

## Methods

Our estimation strategy exploits cross-city variation in the increase in handgun purchasing from the election of President Obama until six weeks post Sandy Hook to estimate the within-city change in the rate of firearm-related harm associated with the additional handguns acquired during this period.

Our unit of analysis is the city, defined as localities with a population of 10,000 or greater. Because injury data are only available at the zip code level, to maintain geographic parity across our measures, all data were collected at the zip code level and aggregated to corresponding cities. Our sample includes a total of 499 California cities.

Our exposure of interest is the estimated number of city-level excess handguns purchased beginning with the election of President Obama until six weeks following Sandy Hook. The spike (defined below) in firearm purchasing following both events is well-documented, nationally and specifically in California (Levine & McKnight, 2017; Liu & Wiebe, 2019; Studdert et al., 2017; Wallace, 2015). Both events were highly publicized so that the level of exposure to each event across California is arguably uniform, while the handgun purchasing behavior following the events is not. If true, this limits the potential for confounding bias due to factors correlated with firearm purchasing. Since 1953, California has required licensed retailers to generate a separate record for every handgun transfer. These dealer’s records of sale are stored by the California Department of Justice and were made available for use in this study. As all legal transfers of firearms in California must be conducted through a licensed retailer with few exceptions, the database of sales records constitutes a nearly complete record of legal handgun transfers in the state.

Our outcomes include firearm mortality, firearm injury, and each broken down by type (interpersonal, self-directed, unintentional, and undetermined). Firearm fatalities are measured using death records from the California Department of Public Health Vital Records. Nonfatal firearm hospitalizations and emergency department visits are provided by the Office of Statewide Health Planning and Development (OSHPD).

We define excess handguns as the difference between actual handgun acquisitions and expected acquisitions, as predicted by seasonal autoregressive integrated moving-average (SARIMA) time series models, over an 11-week period beginning from the election on November 6, 2012 until 6 weeks post Sandy Hook. The length of our spike period is based on Studdert et al’s (2017) finding that handgun acquisitions were higher than expected following the 2012 election, increased sharply again immediately following Sandy Hook, and then reverted to expected levels approximately 6 weeks later. We fit SARIMA models to each city’s purchasing time series using the Hyndman and Khandakar algorithm (Hyndman & Khandakar, 2008). We tested for residual autocorrelation using the Box-Ljung test (Ljung & Box, 1978), with the false discovery rate multiple test correction of Benjamini and Hochberg (Benjamini & Hochberg, 1995). One city was found to have residual autocorrelation, which was corrected by including a seasonal differencing term. Using time binned in 73-day periods (approximately 11 weeks), the training period for the SARIMA models covers January 1, 2004 – November 5, 2012.

We then use Poisson regression models to estimate the effects of city-level excess handgun acquisitions on city-level changes in rates of firearm mortality and injury and by type in 11-week, 6-month, and 1-year windows following the election, as compared with the same time periods directly before the election. Outcomes were not assessed until 10 days after the election, the first day an election-day purchaser could legally aquire the handgun by California law. We include fixed effects for time to account for temporal  trends in firearm violence and for city to adjust for time-fixed differences across place. We thereby estimate the impact of acquisitions on firearm-related harm based on within-city variation above and beyond city averages for the dependent and explanatory variables.

To test the robustness of our results, we estimate several alternative specifications including binning purchases in shorter time periods (1-week and 6-week bins) in our SARIMA models. We also incorporate uncertainty from the first stage model (the estimate of the spike size) in our second stage models by estimating effects using the minimum and maximum bounds of the 95% prediction interval for the spike size. Finally, we implement several falsification tests, estimating models with a set of outcomes that should not be affected by the exposure (handgun purchasing). Specifically, we test for an “effect” on bicycle injury (involving a hospitalization) and machinery injury, both of which have a similar statewide base rate to firearm injury, and on motor vehicle crash injury, often cited in the firearm literature as an example of a successful application of the public health approach to reducing injury and mortality (Hemenway, 2010; Hemenway, 2001). Because the base rate for motor vehicle crashes is 33 times greater than firearm injuries, we scale the outcome so as to have the same power to detect statistical significance.

## Results

**Fig. 1:**
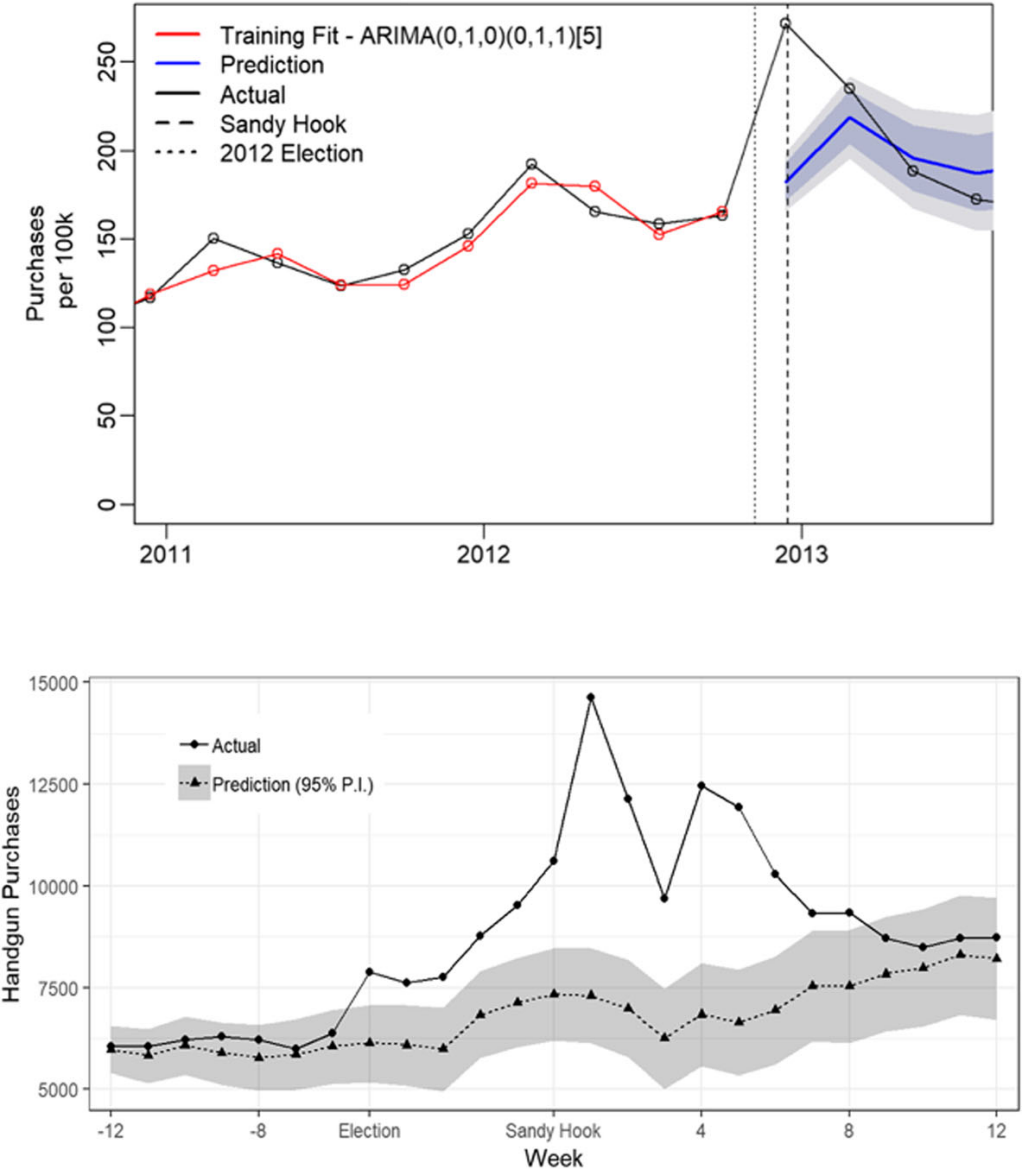
Statewide handgun purchasing. Weekly handgun acquisitions per 100,000, 2011- 2015 top figure. Number of handgun acquisitions per week October 2014 – January 2015 bottom figure

Using SARIMA model predictions of excess purchasing at the city level, we estimate a total of 36,142 (95% prediction interval: 22,780 to 49,505) excess handgun acquisitions in California from the election through the 6-week period following Sandy Hook, representing an increase of more than 55% over expected volume. Figure 1 shows the statewide handgun acquisition spike. Importantly, expected acquisitions closely approximate actual acquisitions during the pre-election period. Additional file 1: Figure S1 shows the size and locations of estimated purchasing spikes.

The Poisson regression models show city-level purchasing spikes are statistically significantly associated with higher rates of firearm injury in the year following the election. Specifically, we estimate one additional excess handgun purchase per 1,000 people is associated with 1.044 times the injury rate (1.044; 95% CI: 1.000, 1.089) (Table 1). Using model predictions, we estimate a total of approximately 290 (95% CI: 0 to 616) additional firearm injuries occurred in California over a year in connection to the excess handguns purchased in the 11 weeks following the election, a statewide increase of nearly 4% (95% CI: 0, 8%). We also find a statistically significant association specifically with interpersonal injury (1.092; 95% CI: 1.003, 1.189) (Additional file 1: Table S1). Exploratory analysis suggests the difference in interpersonal injury is the main driver of the overall difference in firearm injury.

**Table 1:** Association between excess handguns (per 1,000 population) acquired during the 11 week period following the 2012 election and Sandy Hook and firearm violence rates (per 1,000 Population)

outcome window	all firearm violence	firearm injury	firearm mortality	machinery injury (falsification test)	bicycle injury (falsification test)	motor vehicle injury (falsification test)
*Poisson* *Regression* *Risk* *Ratios* ^*†*^						
12 Month	1.033	1.044 *	0.993	1.014	0.956	0.988
	(0.997, 1.070)	**(1.000, 1.089)**	(0.932, 1.058)	(0.998, 1.031)	(0.897, 1.019))	(0.954, 1.023)

* *P* ≤ 0.05

^†^ Results for injury and mortality by type and for shorter time windows are included in the Additional file 1

We do not detect a statistically significant difference for the shorter time periods or firearm mortality. Using the base rate of the 1-year injury estimate, scaled to 11 weeks, 6 months, and 1 year, we conduct post hoc simulations to evaluate our power to detect statistically significant associations. Running 1,000 simulations we find our power to detect an association in the 11 week window is only 0.16, as compared to 0.52 for the 1-year period. Simulation results are presented in the Additional file 1: Figure S2.

The results from all of our robustness checks support the finding of an association with firearm injury, with one exception. Results are substantively similar using excess purchasing estimates from the 1-week and 6-week bins for the first stage SARIMA models, but are only statistically significant for the 6-week bins (Additional file 1: Table S2). Incorporating uncertainty from the first stage SARIMA models (upper and lower bound confidence interval), results for firearm injury remain statistically significant (Additional file 1: Table S3). The falsification test outcomes all have risk ratios that are not significantly different from 1 (Table 1).

## Discussion

Our results suggest that cities that experienced substantial increases in the rate of handgun purchasing in the wake of Obama’s reelection and the Sandy Hook school shooting were more likely to see an increase in the rate of firearm injury the following year. We estimated an increase in injuries of 4% over the following year for the entire state. This 4% represents an important increase in the total number of people injured – approximately 290 additional firearm injuries in California. A similar study of effects of the post-Sandy Hook purchasing spike on firearm mortality in the United States estimated there were approximately 137 additional accidental firearm deaths in the entire country associated with the excess firearms (Levine & McKnight, 2017).

Results were somewhat dependent on the length of time used to bin purchases, which influenced the estimate of the number of excess handgun purchases across cities. Final results were not significant for the models that used 1-week bins to estimate the spike size. These estimates were less stable, particularly for small cities. The magnitude of the estimated association between purchasing spikes and firearm harm was largest for models using the 6-weeks bin estimates (1.066; 95% CI: 1.024, 1.110), though these bins included some post-spike time beyond the ~ 11 week spike period.

We do not see effects for firearm mortality or for shorter time periods (11 weeks and 6 months). The only other study to estimate the effects of purchasing spikes on firearm mortality also did not find associations for firearm suicide or homicide (Levine & McKnight, 2017). Given the baseline prevalence of firearm ownership is already high and the mortality outcomes are relatively rare, we would not expect to see a large population level effect of the spike in handgun acquisitions. Though the purchasing spike was substantial, it accounted for less than 10% of annual handgun acquisitions, and is a tiny fraction of the more than 30 million estimated privately owned firearms in California (Okoro et al., 2005). We also found our power to detect associations for shorter time periods and the rarer outcomes was quite low. As a result, we cannot confirm whether the failure to identify additional associations was due to a true lack of association or low power to detect an association.

This is the first study to use a direct measure of handgun purchasing to estimate the association between firearm acquisitions and firearm-related harm. It is also the first to assess impacts on firearm injury. Additionally, the use of spikes in purchasing following the 2012 presidential election and the Sandy Hook school shooting to identify city-level influxes of handguns is a novel approach that minimizes the potential for confounding. To bias the association, the presidential election, Sandy Hook, or another closely timed historical event would need to influence firearm purchasing behavior and, independently from purchasing behavior, rates of firearm harm in the same geographic areas. Finally, three falsification tests using outcomes that we would not expect to be associated with firearm purchasing or the events preceding the purchasing spike showed no association with the number of excess handguns.

An important limitation to be noted is that we can only empirically test for relatively short-term (≤1 year) associations: moving further in time from the event of interest raises the possibility that other factors drove the witnessed differences in city-level firearm harm. Yet the risks associated with having a handgun in the home are clearly not temporally limited. As such, there may be cumulative and long-term effects of excess handgun purchases that are simply not detectable with this data and design. Similarly, while our finding of increased injury is credibly the result of increased exposure to handguns generated by the purchasing spike, the study design does not rule out the possibility that cities that had more purchasing following the election and Sandy Hook were also cities that have done less to reduce community level violence in the post-spike period. Finally, injury data are available at the zip code but not city level and there may have been some misattribution to place in our conversion from zip code to city data. However, the same geographic areas were used to measure both our exposure and outcomes so this should not have biased our results.

## Conclusion

Firearm ownership is consistently associated with an increased risk of firearm harm at the individual and population levels (Siegel et al., 2013; Wiebe, 2003). Here, we find evidence that cities with larger spikes in handgun acquisitions following the 2012 presidential election had relatively higher rates of firearm-related injury the following year. This suggests that even marginal increases in handgun prevalence may be impactful. These results require replication elsewhere and an assessment of threshold effects in the relationship between firearm prevalence and firearm harm.

## Additional file

Additional file 1: **Figure S1.** Heat map of city spikes. **Figure S2.** Power calculation simulations. **Table S1.** All time periods and all outcomes. **Table S2.** Risk Ratios from SARIMA models using 1-week and 6-week bins (52 week results). **Table S3.** Risk ratios calculated from lower and upper bounds of first stage estimates. (PDF 490 kb)

## Data Availability

The data that support this study are not publicly available, but can be obtained from the California Department of Justice and the State Office of Statewide Health Planning and Development.

## Abbreviations

CI: Confidence Interval
NICS: National Instant Criminal Background Check System
SARIMA: Seasonal autoregressive integrated moving-average
