# Supplementary material for: The impact of spikes in handgun acquisitions on firearm-related harms
Source: Inj Epidemiol. 2019 Aug 26;6:35. doi: 10.1186/s40621-019-0212-0 (PMC6709554; doi:10.1186/s40621-019-0212-0)
Supplement: Supplementary file 1 — Figure S1. Heat map of city spikes. Figure S2. Power calculation simulations. Table S1. All time periods and all outcomes. Table S2. Risk Ratios from SARIMA models using 1-week and 6-week bins (52 week results). Table S3. Risk ratios calculated from lower and upper bounds of first stage estimates. (PDF 490 kb) [file 40621_2019_212_MOESM1_ESM.pdf]

**SUPPLEMENTARY MATERIAL**
For
The Impact of Spikes in Handgun Acquisitions on Firearm-Related Harms

Hannah S. Laqueur[CO] PhD, MS, MPA, Rose M.C. Kagawa[CO] PhD, MPH, Christopher D. McCort MS, Rocco Pallin MPH, Garen Wintemute MD, MPH

*CO indicates co-first authorship

Table of Contents

Figure S1: Heat map of city spikes

Figure S2: Power calculation simulations

Table S1: All time periods and all outcomes

Table S2: Risk Ratios from SARIMA models using 1-week and 6-week bins (52-week results)

Table S3: Risk ratios calculated from lower and upper bounds of first stage estimates

### FIGURE S1: HEAT MAP OF CITY SPIKES

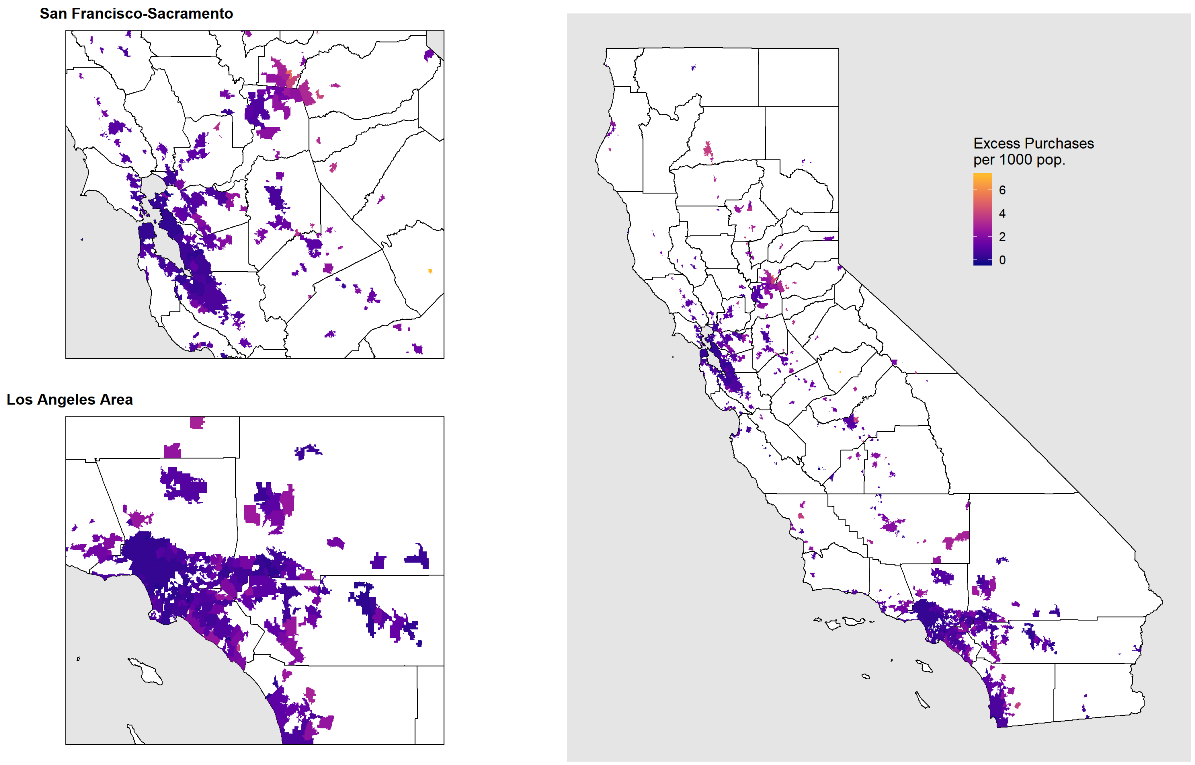

## FIGURE S2: POWER CALCULATION SIMULATIONS[1,2]

### INJURY (EXCLUDING MORTALITY)

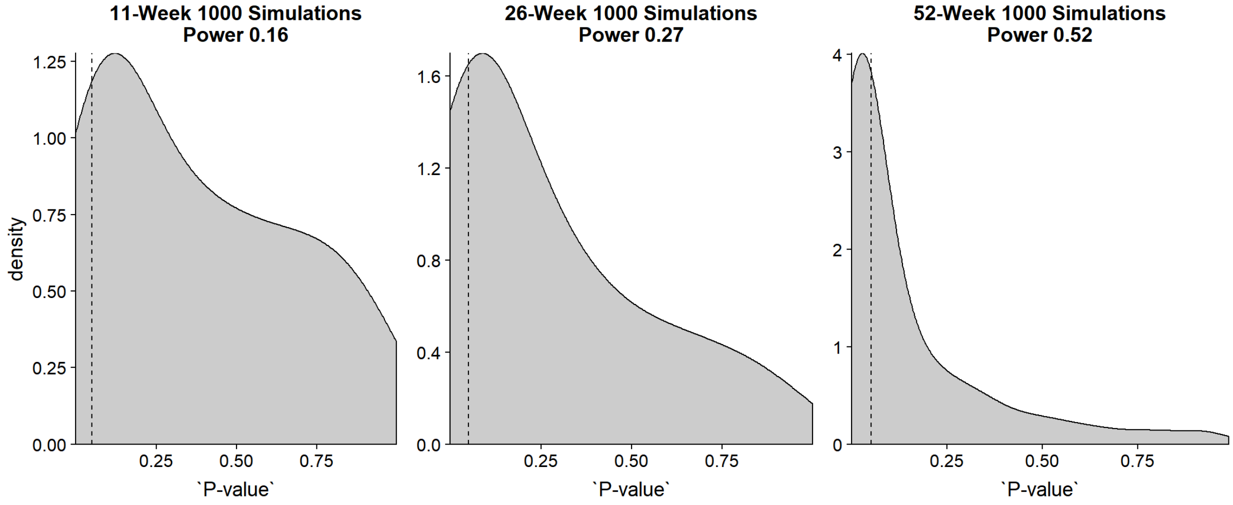


### MORTALITY

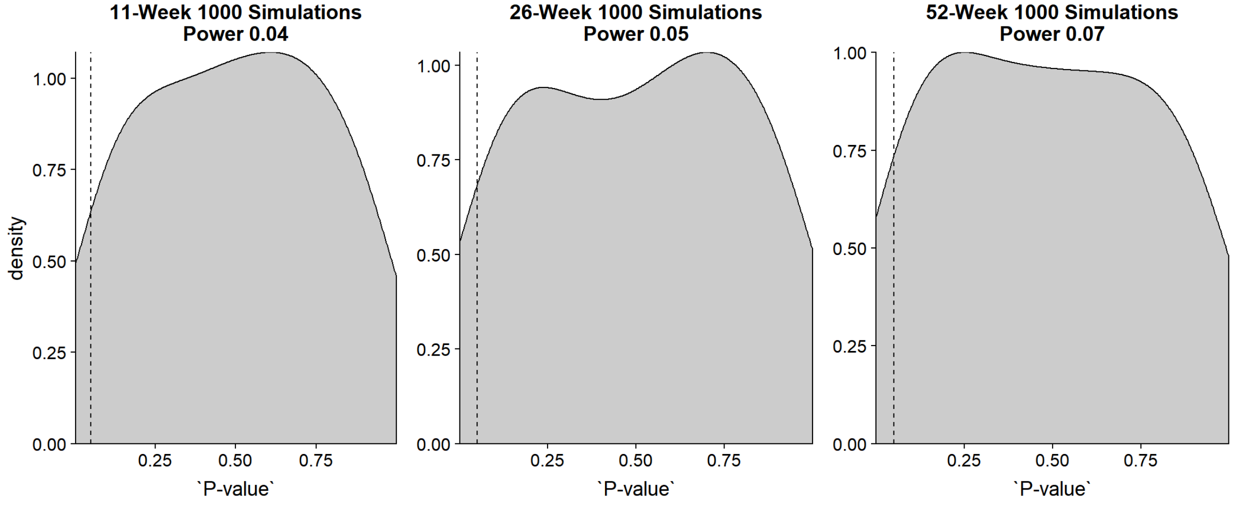


[1] Power is calculated as the area under the curve from [0, 0.05]
[2] Densities are smoothed

**TABLE S1: ALL TIME PERIODS AND ALL OUTCOMES**

| | **11-Week** Risk Ratio (95% CI) | **26-Week** Risk Ratio (95% CI) | **52-Week** Risk Ratio (95% CI) |
|---|---|---|---|
| **Firearm Violence** | 1.018 | 1.029 | 1.033 |
| | (0.945, 1.097) | (0.980, 1.081) | (0.997, 1.070) |
| **Firearm Mortality** | 1.077 | 1.042 | 0.993 |
| | (0.936, 1.242) | (0.953, 1.139) | (0.932, 1.058) |
| **Interpersonal** | 0.967 | 0.977 | 0.937 |
| | (0.771, 1.206) | (0.837, 1.140) | (0.837, 1.049) |
| **Self-directed** | 1.137 | 1.033 | 1.001 |
| | (0.928, 1.396) | (0.916, 1.165) | (0.921, 1.088) |
| **Unintentional** | 0.333 | 0.794 | 0.765 |
| | (0.091, 0.879) | (0.397, 1.539) | (0.442, 1.279) |
| **Undetermined** | 1.884 | 3.619 | 0.656 |
| | (0.000, Inf) | (0.690, 112.811) | (0.187, 1.781) |
| **Firearm Injury** | 0.985 | 1.012 | 1.044 |
| | (0.901, 1.076) | (0.953, 1.074) | (1.000, 1.089) |
| **Interpersonal** | 1.092 | 1.119 | 1.092 |
| | (0.920, 1.296) | (0.997, 1.256) | (1.003, 1.189) |
| **Self-directed** | 0.9 | 0.944 | 1.099 |
| | (0.454, 1.709) | (0.624, 1.414) | (0.860, 1.407) |
| **Unintentional** | 1.135 | 0.994 | 1.057 |
| | (0.799, 1.608) | (0.775, 1.270) | (0.892, 1.252) |
| **Undetermined** | 0.253 | 0.958 | 1.185 |
| | (0.032, 0.958) | (0.554, 1.651) | (0.778, 1.817) |

Associations between excess handguns (per 1,000 population) acquired during the 11-week period following the 2012 presidential election and Sandy Hook and firearm violence rates (per 1,000 population)

**TABLE S2: RISK RATIOS FROM SARIMA MODELS USING 1-WEEK AND 6-WEEK BINS (52-WEEK ESTIMATES)**

| | **1-Week Bins** Risk Ratio (95% CI) | **6-Week Bins** Risk Ratio (95% CI) |
|---|---|---|
| **Firearm Violence** | 1.022 (0.991, 1.053) | 1.045 (1.012, 1.079) |
| **Firearm Mortality** | 0.985 (0.932, 1.042) | 0.992 (0.937, 1.050) |
| **Firearm Injury** | 1.032 (0.994, 1.071) | 1.066 (1.024, 1.110) |

Purchases are binned in shorter time periods (1-week and 6-week bins) in SARIMA models
1-week bin estimates were less stable, particularly for small cities
6-week bins included some post-spike time in the spike period

**TABLE S3: RISK RATIOS CALCULATED FROM LOWER AND UPPER BOUNDS OF FIRST STAGE ESTIMATES**

| | 52-Week Estimates | Estimates Using Lower Bound (of spike) from First Stage | Estimates Using Upper Bound (of spike) from First Stage |
|---|---|---|---|
| **Firearm Violence** | 1.033 | 1.039 | 1.027 |
| | (0.997, 1.070) | (0.997, 1.083) | (0.997, 1.058) |
| **Firearm Mortality** | 0.993 | 0.996 | 0.992 |
| | (0.932, 1.058) | (0.925, 1.073) | (0.940, 1.046) |
| **Firearm Injury** | 1.044 | 1.051 | 1.037 |
| | (1.000, 1.089) | (1.000, 1.104) | (1.000, 1.076) |
